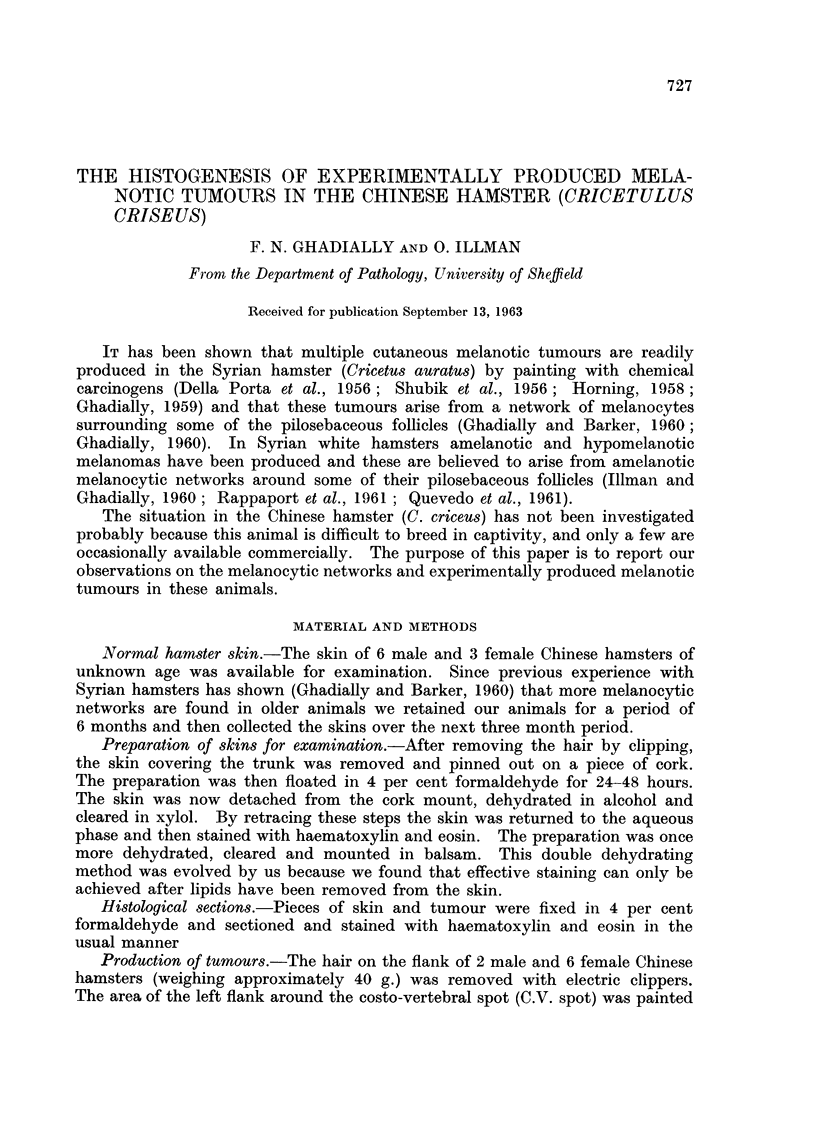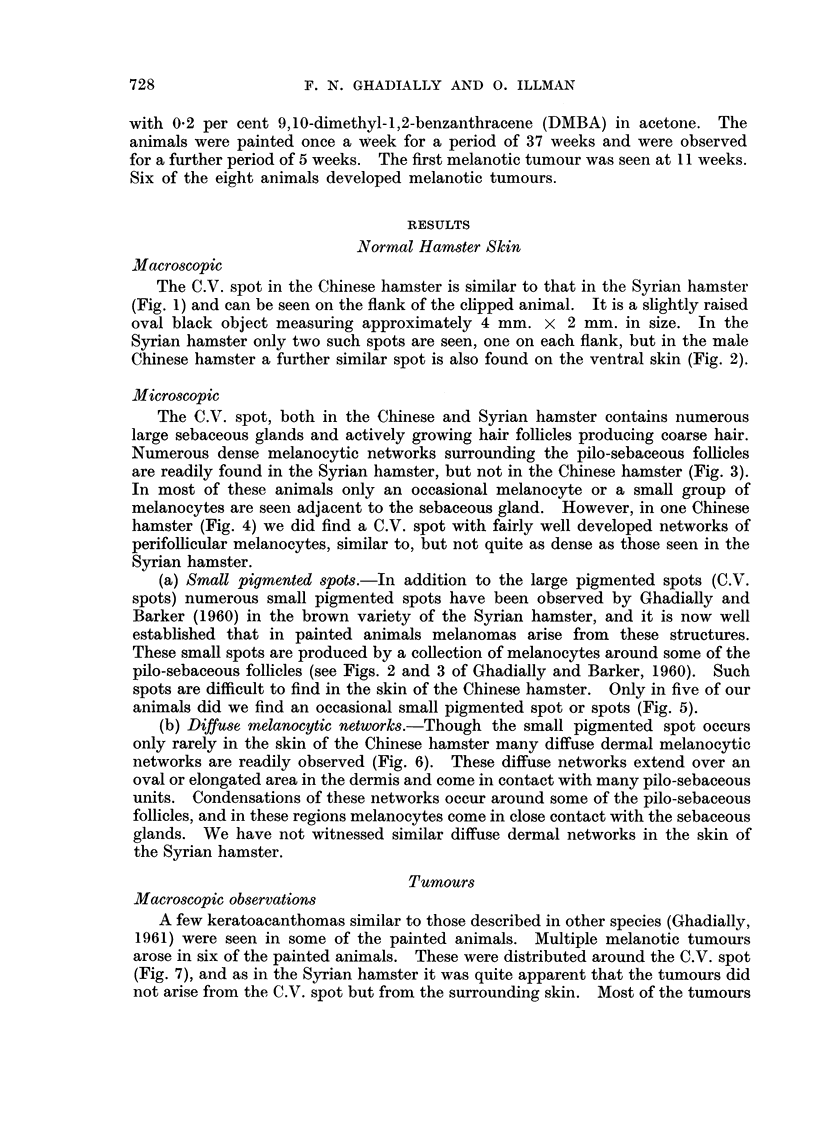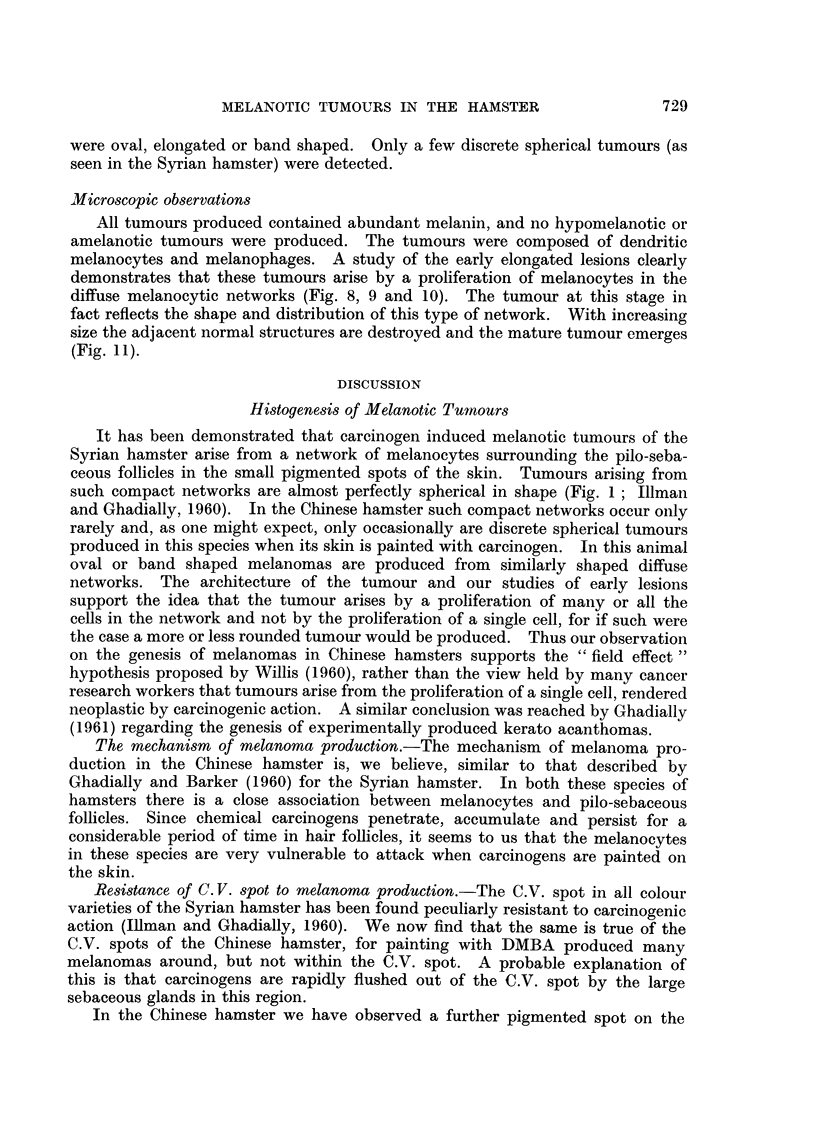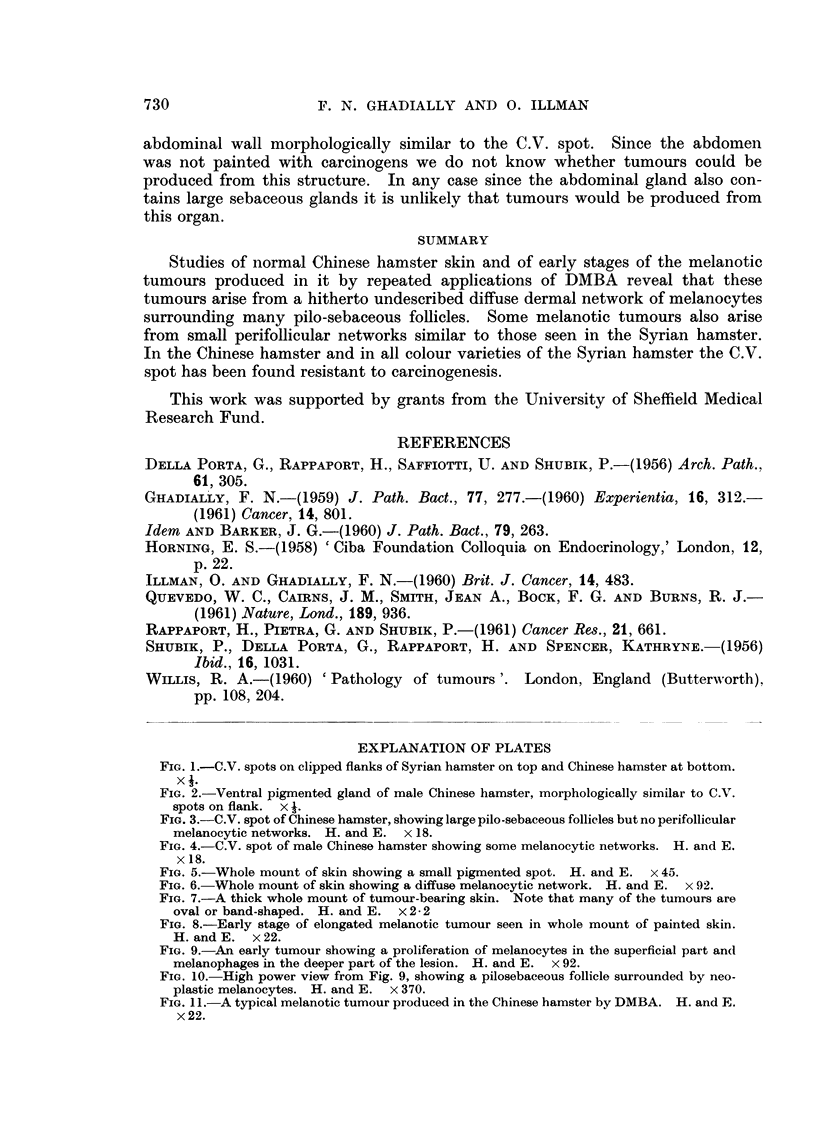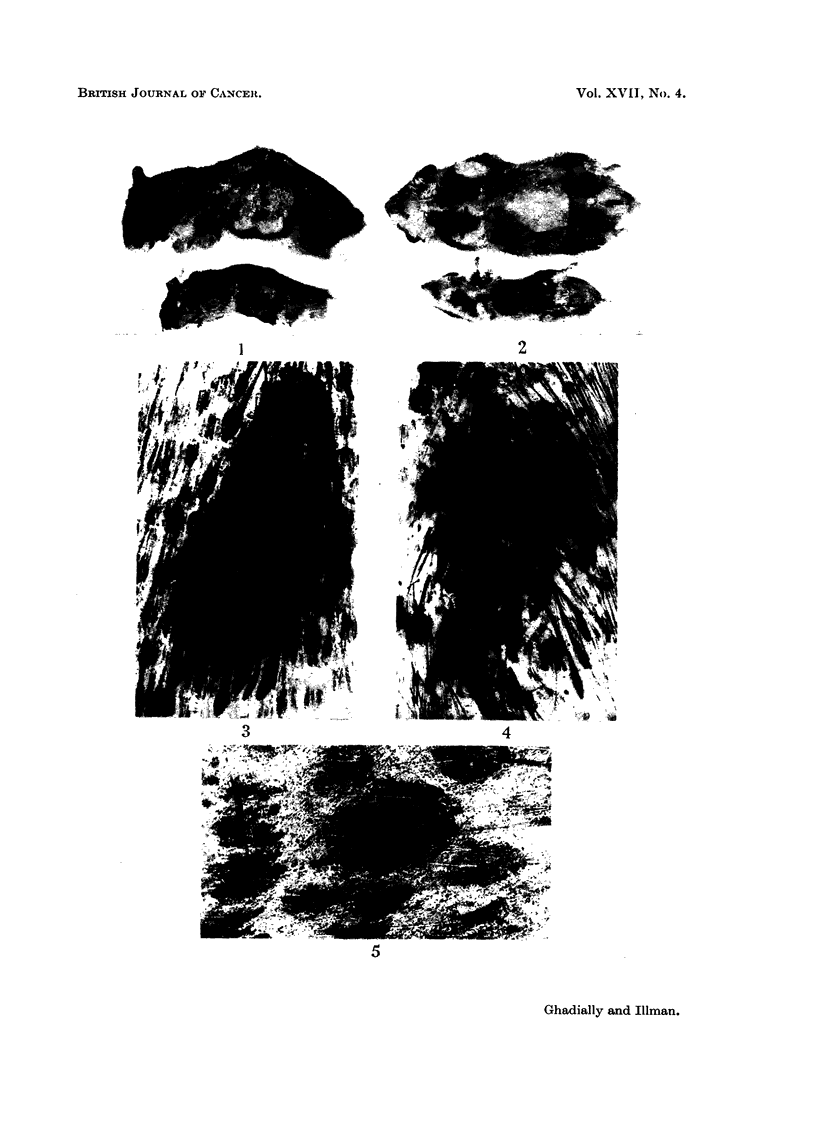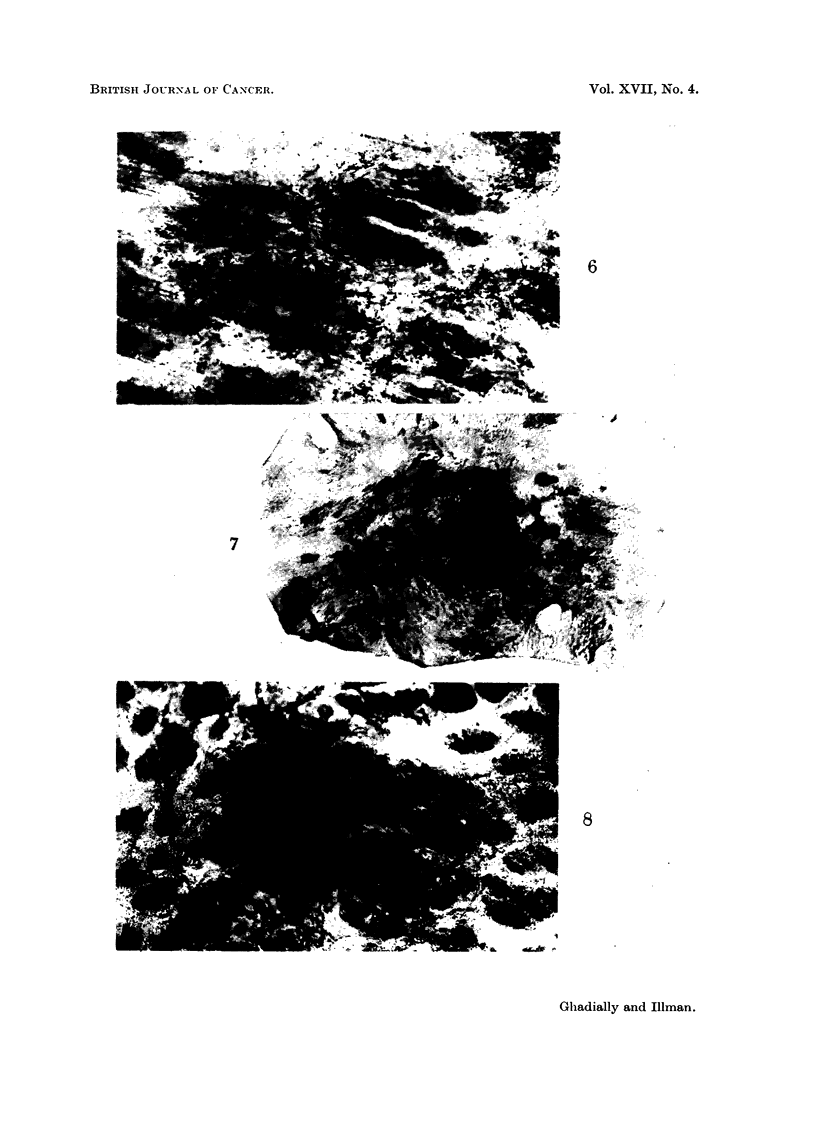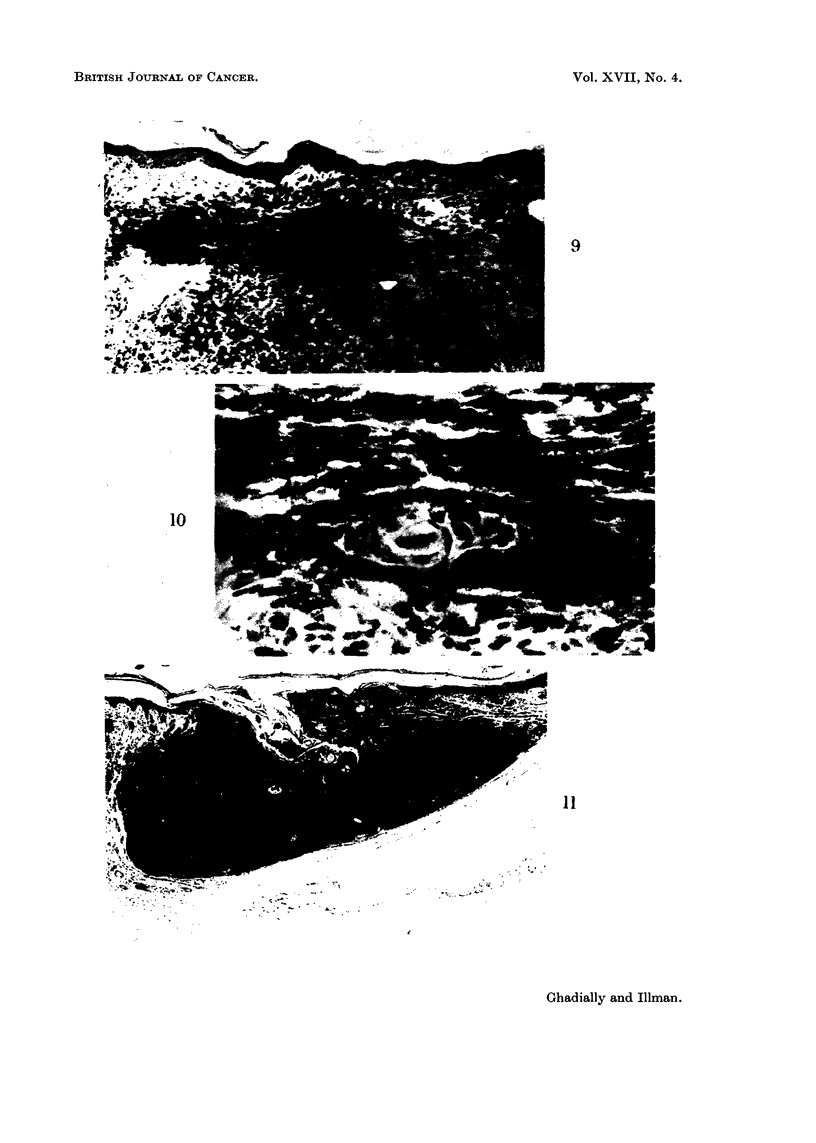# The Histogenesis of Experimentally Produced Melanotic Tumours in the Chinese Hamster (Cricetulus Criseus)

**DOI:** 10.1038/bjc.1963.93

**Published:** 1963-12

**Authors:** F. N. Ghadially, O. Iliman

## Abstract

**Images:**


### THE HISTOGENESIS OF EXPERIMENTALLY PRODUCED MELANOTIC TUMOURS IN THE CHINESE HAMSTER (*CRICETULUS CRISEUS*)

F. N. GHADIALLY AND O. ILLMAN

*From the Department of Pathology, University of Sheffield*

Received for publication September 13, 1963

IT has been shown that multiple cutaneous melanotic tumours are readily produced in the Syrian hamster (*Cricetus auratus*) by painting with chemical carcinogens (Della Porta *et al.*, 1956 ; Shubik *et al.*, 1956 ; Horning, 1958 ; Ghadially, 1959) and that these tumours arise from a network of melanocytes surrounding some of the pilosebaceous follicles (Ghadially and Barker, 1960 ; Ghadially, 1960). In Syrian white hamsters amelanotic and hypomelanotic melanomas have been produced and these are believed to arise from amelanotic melanocytic networks around some of their pilosebaceous follicles (Illman and Ghadially, 1960 ; Rappaport *et al.*, 1961 ; Quevedo *et al.*, 1961).

The situation in the Chinese hamster (*C. criceus*) has not been investigated probably because this animal is difficult to breed in captivity, and only a few are occasionally available commercially. The purpose of this paper is to report our observations on the melanocytic networks and experimentally produced melanotic tumours in these animals.

### MATERIAL AND METHODS

*Normal hamster skin.*—The skin of 6 male and 3 female Chinese hamsters of unknown age was available for examination. Since previous experience with Syrian hamsters has shown (Ghadially and Barker, 1960) that more melanocytic networks are found in older animals we retained our animals for a period of 6 months and then collected the skins over the next three month period.

*Preparation of skins for examination.*—After removing the hair by clipping, the skin covering the trunk was removed and pinned out on a piece of cork. The preparation was then floated in 4 per cent formaldehyde for 24–48 hours. The skin was now detached from the cork mount, dehydrated in alcohol and cleared in xylol. By retracing these steps the skin was returned to the aqueous phase and then stained with haematoxylin and eosin. The preparation was once more dehydrated, cleared and mounted in balsam. This double dehydrating method was evolved by us because we found that effective staining can only be achieved after lipids have been removed from the skin.

*Histological sections.*—Pieces of skin and tumour were fixed in 4 per cent formaldehyde and sectioned and stained with haematoxylin and eosin in the usual manner

*Production of tumours.*—The hair on the flank of 2 male and 6 female Chinese hamsters (weighing approximately 40 g.) was removed with electric clippers. The area of the left flank around the costo-vertebral spot (C.V. spot) was painted with 0·2 per cent 9,10-dimethyl-1,2-benzanthracene (DMBA) in acetone. The animals were painted once a week for a period of 37 weeks and were observed for a further period of 5 weeks. The first melanotic tumour was seen at 11 weeks. Six of the eight animals developed melanotic tumours.

### RESULTS

### *Normal Hamster Skin*

*Macroscopic*

The C.V. spot in the Chinese hamster is similar to that in the Syrian hamster (Fig. 1) and can be seen on the flank of the clipped animal. It is a slightly raised oval black object measuring approximately 4 mm. $\times$ 2 mm. in size. In the Syrian hamster only two such spots are seen, one on each flank, but in the male Chinese hamster a further similar spot is also found on the ventral skin (Fig. 2).

*Microscopic*

The C.V. spot, both in the Chinese and Syrian hamster contains numerous large sebaceous glands and actively growing hair follicles producing coarse hair. Numerous dense melanocytic networks surrounding the pilo-sebaceous follicles are readily found in the Syrian hamster, but not in the Chinese hamster (Fig. 3). In most of these animals only an occasional melanocyte or a small group of melanocytes are seen adjacent to the sebaceous gland. However, in one Chinese hamster (Fig. 4) we did find a C.V. spot with fairly well developed networks of perifollicular melanocytes, similar to, but not quite as dense as those seen in the Syrian hamster.

(a) *Small pigmented spots.*—In addition to the large pigmented spots (C.V. spots) numerous small pigmented spots have been observed by Ghadially and Barker (1960) in the brown variety of the Syrian hamster, and it is now well established that in painted animals melanomas arise from these structures. These small spots are produced by a collection of melanocytes around some of the pilo-sebaceous follicles (see Figs. 2 and 3 of Ghadially and Barker, 1960). Such spots are difficult to find in the skin of the Chinese hamster. Only in five of our animals did we find an occasional small pigmented spot or spots (Fig. 5).

(b) *Diffuse melanocytic networks.*—Though the small pigmented spot occurs only rarely in the skin of the Chinese hamster many diffuse dermal melanocytic networks are readily observed (Fig. 6). These diffuse networks extend over an oval or elongated area in the dermis and come in contact with many pilo-sebaceous units. Condensations of these networks occur around some of the pilo-sebaceous follicles, and in these regions melanocytes come in close contact with the sebaceous glands. We have not witnessed similar diffuse dermal networks in the skin of the Syrian hamster.

### *Tumours*

*Macroscopic observations*

A few keratoacanthomas similar to those described in other species (Ghadially, 1961) were seen in some of the painted animals. Multiple melanotic tumours arose in six of the painted animals. These were distributed around the C.V. spot (Fig. 7), and as in the Syrian hamster it was quite apparent that the tumours did not arise from the C.V. spot but from the surrounding skin. Most of the tumours were oval, elongated or band shaped. Only a few discrete spherical tumours (as seen in the Syrian hamster) were detected.

*Microscopic observations*

All tumours produced contained abundant melanin, and no hypomelanotic or amelanotic tumours were produced. The tumours were composed of dendritic melanocytes and melanophages. A study of the early elongated lesions clearly demonstrates that these tumours arise by a proliferation of melanocytes in the diffuse melanocytic networks (Fig. 8, 9 and 10). The tumour at this stage in fact reflects the shape and distribution of this type of network. With increasing size the adjacent normal structures are destroyed and the mature tumour emerges (Fig. 11).

### DISCUSSION

### *Histogenesis of Melanotic Tumours*

It has been demonstrated that carcinogen induced melanotic tumours of the Syrian hamster arise from a network of melanocytes surrounding the pilo-sebaceous follicles in the small pigmented spots of the skin. Tumours arising from such compact networks are almost perfectly spherical in shape (Fig. 1 ; Illman and Ghadially, 1960). In the Chinese hamster such compact networks occur only rarely and, as one might expect, only occasionally are discrete spherical tumours produced in this species when its skin is painted with carcinogen. In this animal oval or band shaped melanomas are produced from similarly shaped diffuse networks. The architecture of the tumour and our studies of early lesions support the idea that the tumour arises by a proliferation of many or all the cells in the network and not by the proliferation of a single cell, for if such were the case a more or less rounded tumour would be produced. Thus our observation on the genesis of melanomas in Chinese hamsters supports the " field effect " hypothesis proposed by Willis (1960), rather than the view held by many cancer research workers that tumours arise from the proliferation of a single cell, rendered neoplastic by carcinogenic action. A similar conclusion was reached by Ghadially (1961) regarding the genesis of experimentally produced kerato acanthomas.

*The mechanism of melanoma production.*—The mechanism of melanoma production in the Chinese hamster is, we believe, similar to that described by Ghadially and Barker (1960) for the Syrian hamster. In both these species of hamsters there is a close association between melanocytes and pilo-sebaceous follicles. Since chemical carcinogens penetrate, accumulate and persist for a considerable period of time in hair follicles, it seems to us that the melanocytes in these species are very vulnerable to attack when carcinogens are painted on the skin.

*Resistance of C.V. spot to melanoma production.*—The C.V. spot in all colour varieties of the Syrian hamster has been found peculiarly resistant to carcinogenic action (Illman and Ghadially, 1960). We now find that the same is true of the C.V. spots of the Chinese hamster, for painting with DMBA produced many melanomas around, but not within the C.V. spot. A probable explanation of this is that carcinogens are rapidly flushed out of the C.V. spot by the large sebaceous glands in this region.

In the Chinese hamster we have observed a further pigmented spot on the abdominal wall morphologically similar to the C.V. spot. Since the abdomen was not painted with carcinogens we do not know whether tumours could be produced from this structure. In any case since the abdominal gland also contains large sebaceous glands it is unlikely that tumours would be produced from this organ.

### SUMMARY

Studies of normal Chinese hamster skin and of early stages of the melanotic tumours produced in it by repeated applications of DMBA reveal that these tumours arise from a hitherto undescribed diffuse dermal network of melanocytes surrounding many pilo-sebaceous follicles. Some melanotic tumours also arise from small perifollicular networks similar to those seen in the Syrian hamster. In the Chinese hamster and in all colour varieties of the Syrian hamster the C.V. spot has been found resistant to carcinogenesis.

This work was supported by grants from the University of Sheffield Medical Research Fund.

### REFERENCES

Della Porta, G., Rappaport, H., Saffiotti, U. and Shubik, P.—(1956) *Arch. Path.*, **61**, 305.

Ghadially, F. N.—(1959) *J. Path. Bact.*, **77**, 277.—(1960) *Experientia*, **16**, 312.—(1961) *Cancer*, **14**, 801.

*Idem* and Barker, J. G.—(1960) *J. Path. Bact.*, **79**, 263.

Horning, E. S.—(1958) 'Ciba Foundation Colloquia on Endocrinology,' London, **12**, p. 22.

Illman, O. and Ghadially, F. N.—(1960) *Brit. J. Cancer*, **14**, 483.

Quevedo, W. C., Cairns, J. M., Smith, Jean A., Bock, F. G. and Burns, R. J.—(1961) *Nature, Lond.*, **189**, 936.

Rappaport, H., Pietra, G. and Shubik, P.—(1961) *Cancer Res.*, **21**, 661.

Shubik, P., Della Porta, G., Rappaport, H. and Spencer, Kathryne.—(1956) *Ibid.*, **16**, 1031.

Willis, R. A.—(1960) 'Pathology of tumours'. London, England (Butterworth), pp. 108, 204.

---

### EXPLANATION OF PLATES

Fig. 1.—C.V. spots on clipped flanks of Syrian hamster on top and Chinese hamster at bottom. $\times \frac{1}{3}$.

Fig. 2.—Ventral pigmented gland of male Chinese hamster, morphologically similar to C.V. spots on flank. $\times \frac{1}{2}$.

Fig. 3.—C.V. spot of Chinese hamster, showing large pilo-sebaceous follicles but no perifollicular melanocytic networks. H. and E. ×18.

Fig. 4.—C.V. spot of male Chinese hamster showing some melanocytic networks. H. and E. ×18.

Fig. 5.—Whole mount of skin showing a small pigmented spot. H. and E. ×45.

Fig. 6.—Whole mount of skin showing a diffuse melanocytic network. H. and E. ×92.

Fig. 7.—A thick whole mount of tumour-bearing skin. Note that many of the tumours are oval or band-shaped. H. and E. ×2·2

Fig. 8.—Early stage of elongated melanotic tumour seen in whole mount of painted skin. H. and E. ×22.

Fig. 9.—An early tumour showing a proliferation of melanocytes in the superficial part and melanophages in the deeper part of the lesion. H. and E. ×92.

Fig. 10.—High power view from Fig. 9, showing a pilosebaceous follicle surrounded by neoplastic melanocytes. H. and E. ×370.

Fig. 11.—A typical melanotic tumour produced in the Chinese hamster by DMBA. H. and E. ×22.